# Cholera in Yangon, Myanmar, 2012–2013

**DOI:** 10.3201/eid2103.141309

**Published:** 2015-03

**Authors:** Wah Wah Aung, Kazuhisa Okada, Mathukorn Na-Ubol, Wirongrong Natakuathung, Toe Sandar, Nan Aye Thidar Oo, Mya Mya Aye, Shigeyuki Hamada

**Affiliations:** Ministry of Health Department of Medical Research (Lower Myanmar);; Yangon, Myanmar (W.W. Aung, N.A.T Oo, M.M. Aye);; Thailand-Japan Research Collaboration Center on Emerging and Re-emerging Infections, Nonthaburi, Thailand (K. Okada, M. Na-Ubol, W. Natakuathung, S. Hamada);; Osaka University Research Institute for Microbial Diseases, Osaka, Japan (K. Okada, S. Hamada);; University of Medicine, Yangon (T. Sandar)

**Keywords:** cholera, Myanmar, bacteria, Vibrio cholerae O1, Vibrio cholerae, El Tor, isolates, diarrhea

**To the Editor:** *Vibrio cholerae* O1, a causative agent of cholera, is classified into 2 biotypes, classical and El Tor. Since 1817, cholera has spread from the Indian subcontinent to other regions of the globe 7 times (*1*). However, little information on the occurrence of cholera and *V. cholerae* in Myanmar has been published. Here, we report cholera cases and characterization of 58 clinical isolates of *V. cholerae* O1 serotype Ogawa recovered from patients with diarrhea in Yangon, Myanmar, during February 2012–June 2013.

During February and August 2012, rectal swab specimens were collected from patients suspected of having cholera in 4 hospitals in Yangon: New Yangon General Hospital, North Okkalapa General Hospital, Thingangyun Sanpya General Hospital, and Yangon Children Hospital. The specimens were cultured on thiosulfate citrate bile salts sucrose agar plates. After overnight incubation, several colonies that resembled to those of *V. cholerae* were confirmed as serogroup O1 by using slide agglutination tests with specific monoclonal antibodies (*2*).

Of the tested specimens, 34 isolates carried the *tcpA* gene, encoding the structural subunit of toxin-coregulated pilus, and the *rstR* gene, the repressor gene in the cholera toxin encoding (CTX) phage (*3*); these results may indicate that these strains belonged to the El Tor biotype. However, identification of the sequence type of the cholera toxin B subunit gene in these isolates revealed that they were of classical type (*ctxB*^Cla^). Thus, these isolates were classified as atypical El Tor *V. cholerae* O1 (*4*) carrying *ctxB*^Cla^ and *rstR*^El^. Currently, the predominant clones causing cholera in Asia and Africa are atypical El Tor *V. cholerae*, CIRS101, and CIRS101-like variants (*5*,*6*). Myanmar isolates from 2012 and the CIRS101 strain contained a single nucleotide polymorphism in the *tcpA* gene at nt 266 (A→G) of the prototype seventh pandemic El Tor (N16961) strain.

Pulsed-field gel electrophoresis (PFGE) (*7*) using the 2012 isolates revealed 9 patterns (Table). During the initial phase of cholera occurrences, *V. cholerae* O1 was mainly isolated from adults, and 9 pulsotypes were observed. During the later period (May–August), most isolates were from children <5 years of age, and pulsotype Y6 predominated.

**Table:** Characterization of *Vibrio cholerae* O1 isolates from patients in Myanmar, 2012–2013*

Date of isolation	Hospital	Patient age, y/sex	PFGE	MLVA†
2012 Feb				
10	TSGH	18/M	Y9	10, 6, 6, 17, 11
13	TSGH	24/M	Y1	11, 7, 6, 14, 16
2012 Mar				
13	TSGH	1/M	Y2	10, 6, 6, 16, 17
15	TSGH	12/M	Y4	10, 6, 6, 16, 17
16	TSGH	18/F	Y5	10, 6, 6, 16, 17
19	NOGH	68/M	Y7	11, 6, 6, 17, 17
20	NYGH	24/F	Y6	11, 6, 6, 17, 17
23	NYGH	56/M	Y6	11, 6, 6, 17, 17
26	TSGH	54/M	Y4	10, 6, 6, 17, 18
29	NYGH	62/M	Y4	10, 6, 6, 16, 16
29	NYGH	63/F	Y7	10, 6, 6, 18, 18
2012 Apr				
2	TSGH	18/M	Y3	11, 6, 6, 17, 16
2	NYGH	50/F	Y6	11, 6, 6, 16, 17
2	NYGH	20/M	Y8	10, 6, 6, 17, 11
2	NYGH	28/F	Y6	11, 6, 6, 17, 17
2	NYGH	31/F	Y6	11, 6, 6, 17, 17
2	NYGH	22/F	Y7	11, 6, 6, 18, 17
4	NYGH	35/M	Y6	10, 6, 6, 17, 17
4	TSGH	48/F	Y6	11, 6, 6, 17, 17
4	TSGH	17/F	Y7	11, 6, 6, 18, 17
2012 May				
2	YCH	<1/F	Y6	11, 6, 6, 17, 17
7	YCH	4/M	Y6	11, 6, 6, 17, 18
14	YCH	2/M	Y6	11, 6, 6, 17, 18
15	YCH	2/M	Y6	11, 6, 6, 17, 17
2012 Jun				
5	YCH	5/F	Y6	11, 6, 6, 17, 17
5	YCH	2/F	Y6	11, 6, 6, 17, 18
6	YCH	2/F	Y6	11, 6, 6, 17, 18
6	YCH	4/M	Y6	11, 6, 6, 17, 17
14	NOGH	24/F	Y6	11, 6, 6, 17, 17
28	YCH	1/F	Y8	10, 6, 6, 19, 19
2012 Jul				
4	YCH	1/M	Y6	11, 6, 6, 17, 18
30	YCH	5/F	Y7	11, 6, 6, 18, 17
2012 Aug				
6	NOGH	56/F	Y7	11, 6, 6, 17, 18
30	YCH	2/M	Y6	10, 6, 6, 17, 17
2013 Mar				
20	YCH	3/M	Y7	10, 6, 6, 18, 20
2013 Apr				
24	YCH	4/M	Y7	10, 6, 6, 18, 19
30	YCH	1/F	Y7	10, 6, 6, 18, 19
2013 May				
13	YCH	<1/F	Y7	10, 6, 6, 18, 19
20	YCH	4/M	Y7	10, 6, 6, 18, 19
22	YCH	3/M	Y7	10, 6, 6, 18, 20
2013 Jun				
4	YKCH	6/M	Y7	10, 7, 6, 3, 19
6	YCH	1/M	Y7	10, 7, 6, 18, 19
10	YCH	8/M	Y7	10, 6, 6, 17, 19
14	YCH	12/M	Y7	10, 6, 6, 18, 19
14	YCH	9/M	Y7	10, 6, 6, 17, 21
14	YCH	11/M	Y7	10, 6, 6, 17, 19
14	TSGH	1/F	Y7	10, 6, 6, 18, 19
14	YKCH	7/F	Y7	10, 6, 6, 17, 19
14	TSGH	11/M	Y7	10, 6, 6, 17, 19
16	TSGH	11/M	Y7	10, 6, 6, 17, 19
17	TSGH	11/F	Y10	10, 6, 6, 17, 19
19	YCH	1/M	Y7	10, 6, 6, 17, 19
20	YCH	7/M	Y7	10, 6, 6, 17, 19
22	YCH	7/M	Y7	10, 6, 6, 17, 19
24	IGH	<1/F	Y7	10, 6, 6, 17, 19
26	YCH	3/F	Y7	10, 6, 6, 20, 19
26	YCH	1/F	Y7	10, 6, 6, 19, 19
27	IGH	<1/F	Y7	10, 6, 6, 17, 19

*PFGE, pulsed-field gel electrophoresis; MLVA, multilocus variable-number tandem-repeat analysis; TSGH, Thingangyun Sanpya General Hospital; NOGHl North Okkalapa General Hospital; NYGH, New Yangon General Hospital; YCH; Yangon Children Hospital; YKCH, Yankin Children Hospital; IGH, Insein General Hospital. †Numbers of repeats in the 5 genomic variable-number tandem-repeat loci (VC0147, VC0436–7, VC1650, VC0171, and VCA0283).

We carried out multilocus variable-number tandem-repeat analysis (MLVA) (*8*) of the 2012 isolates to resolve distinct populations. MLVA yielded 13 isolate types, and all 18 isolates of pulsotype Y6 exhibited either MLVA profile 11.6.6.17.17 or a closely related profile that differed only by 1 repeat number. These data suggest that cholera was contracted mainly in adults and was caused by multiclonal *V. cholerae* O1. However, in children, *V. cholerae* has transformed from single clonal expansion since May 2012.

During March–June 2013, we extended our studies to characterize cholera organisms isolated from patients with severe diarrhea who were admitted to the original 4 hospitals as well as 2 additional hospitals, Yankin Children Hospital and Insein General Hospital. Of 24 cases, 16 patients showed symptoms of severe dehydration, including 1 patient who experienced shock. Other common symptoms in this patient population included fever (50%, 12/24), vomiting (92%, 22/24), and abdominal pain (33%, 8/24). Although fever is less common among patients with cholera-associated diarrhea (*9*), the frequency of fever was considerably high in this study. PFGE reveled 23 of the 24 isolates were identical to pulsotype Y7, which was the second-most prevalent pattern in 2012; MLVA profiles were also similar to those from 2012. Thus, the occurrences of cholera in 2013 may have been related to persistent transmission of a clone from 2012.

According to surveillance records from the Yangon Regional Health Center, the reported number of diarrhea cases in Yangon increased from 11,651 in 2010 and 11,016 in 2011 to 15,540 in 2012 and 13,919 in 2013. Although there were no reports of cholera outbreaks in Yangon, PFGE/MLVA results revealed that most of the cholera cases in this study were caused by isolates belonging to identical or closely related types. Thus, cholera outbreaks could have occurred in Yangon, and the related clone may have persisted.

In Myanmar, the illness rate for severe diarrhea is estimated to be 2.6–3.5 per 100,000 persons and the mortality rate is 0.04–0.1 per 100,000 (*10*). In this study, the detection rates of *V. cholerae* O1 in stools from patients with severe diarrhea were 23% (49/213 cases) in 2012 and 14% (35/250 cases) in 2013, respectively. Although our investigation is merely the tip of the iceberg for studies of cholera in Myanmar, our data provide crucial initial insights into the genetic backgrounds of recent Yangon isolates of *V. cholerae* O1. Epidemiologic surveillance linked to laboratory investigations is need to minimize the risk for *V. cholerae* infection in children.
